# Minimally Invasive Video-assisted Mitral Valve Replacement with a Right Chest Small Incision in Patients Aged Over 65 Years

**DOI:** 10.21470/1678-9741-2018-0409

**Published:** 2019

**Authors:** Qiang Chen, Ling-Li Yu, Qi-Liang Zhang, Hua Cao, Liang-Wan Chen, Zhong-Yao Huang

**Affiliations:** 1 Department of Cardiovascular Surgery, Union Hospital, Fujian Medical University, Fuzhou, Fujian, People’s Republic of China.

**Keywords:** Mitral Valve, Pneumothorax, Heart Valve Diseases, Thoracotomy, Subcutaneous Emphysema, Length of Stay

## Abstract

**Objective:**

To analyze and summarize the clinical safety and feasibility of minimally invasive video-assisted mitral valve replacement via a right thoracic minimal incision in patients aged over 65 years.

**Methods:**

The clinical data of 45 patients over 65 years old who had mitral valve disease were analyzed retrospectively from January 2014 to January 2017 at Union Hospital, Fujian Medical University. The patients were divided into two groups; 20 patients in group A, who underwent minimally invasive video-assisted mitral valve replacement via a right thoracic minimal incision, and 25 patients in group B, who underwent conventional mitral valve replacement. We collected and analyzed their relevant clinical data.

**Results:**

The operation was completed successfully in both groups. Compared with group B, group A was clearly superior for postoperative analgesia time, postoperative hospital length of stay, thoracic drainage liquid, blood transfusion, and length of incision. There were no differences between the two groups in postoperative severe complications and mortality. More patients in group B had pulmonary infections and poor incision healing, while more patients in group A had postoperative pneumothorax and subcutaneous emphysema.

**Conclusion:**

In patients aged over 65 years, minimally invasive video-assisted mitral valve replacement with a small incision in the right chest had the same clinical safety and efficacy as the conventional method.

**Table:** 

Abbreviations, acronyms & symbols	
BMI	= Body mass index
CPB	= Cardiopulmonary bypass
EF	= Ejection fraction
GFR	= Glomerular filtration rate
MV	= Mitral valve
NYHA	= New York Heart Association
PG	= Peak gradient
SPSS	= Statistical Package for the Social Sciences
TTE	= Transthoracic echocardiography

## INTRODUCTION

With increasing life expectancy and progressing population ageing, the number of elderly patients (aged over 65 years in China) with mitral valve disease who need surgical treatment is increasing yearly^[1,2]^. Elderly patients with heart valve disease usually are in poor health with declining organ function and reserve function^[3,4]^. Therefore, the incidence of postoperative complications is strongly increased, and perioperative mortality is high^[5,6]^.

Traditional open-chest surgery has the advantage of good exposure of the surgical field, which can markedly shorten cardiac arrest and cardiopulmonary bypass (CPB) times, but also has disadvantages of large surgical wounds, more bleeding, severe pain, and slow postoperative recovery^[7,8]^. Total thoracoscopic and thoracoscopic-assisted cardiac surgery have emerged in recent years^[9-11]^. Because of technical difficulties of such procedures^[12-14]^, the operational techniques and clinical effects have not differed from those of traditional open-heart surgery, except when performed in a few specific heart centers, where thoracoscopic surgery is performed early and state-of-the-art technology is available^[15,16]^. Traditional open-chest surgery is currently still used in most centers for elderly patients with mitral valve disease^[17-19]^. The aim of this study was to analyze and summarize the clinical safety and feasibility of minimally invasive video-assisted mitral valve replacement via a right thoracic minimal incision in patients aged over 65 years.

## METHODS

### General Data

A total of 45 patients aged over 65 years with mitral valve disease were analyzed retrospectively from January 2014 to January 2017 at Union Hospital, Fujian Medical University. According to the operative method, the patients were divided into a minimally invasive video-assisted operation group (group A, n=20) and a traditional thoracotomy group, which underwent conventional mitral valve replacement (group B, n=25). All patients had significant clinical symptoms, including palpitations, shortness of breath, and intolerance after exercise. According to the patients’ medical history, symptoms, signs, electrocardiogram, X-ray, and transthoracic echocardiography (TTE), 12 patients were diagnosed with mitral stenosis, five patients with mitral regurgitation, and 28 patients with mitral stenosis and regurgitation. Of these patients, 28 patients had combined moderate or severe tricuspid regurgitation, and six patients had left atrial thrombosis (Table 1).

**Table 1:** Comparison of patients' preoperative general data between the two groups.

Item		Thoracoscopy-assisted group	Conventional group	*P*-value
Age (years)		68.3±2.6	69.1±3.6	0.946
Gender (male/female)		08-Dec	Nov-14	
History of disease (years)		11.4±5.6	13.5±7.3	0.602
BMI		19.1±2.2	18.7±3.1	0.911
NYHA class	I	3	4	0.853
	II	8	8	
	III	9	13	
	IV	0	0	
Mitral lesion type				0.941
Mitral stenosis		5	7	
Mitral regurgitation		2	3	
Mitral stenosis and regurgitation		13	15	
Mitral stenosis	Mild	2	2	0.966
	Moderate	10	13	
	Severe	6	7	
Mitral regurgitation	Mild	1	2	0.730
	Moderate	8	11	
	Severe	6	5	
Moderate or severe tricuspid regurgitation		13	15	0.731
Cardiothoracic ratio		0.67 ±0.06	0.69±0.04	0.909
End-systolic length of the left atrium (mm)		60.7±5.1	63.5±6.2	0.821
End-diastolic length of the left ventricle (mm)		63.5±8.4	65.8±6.7	0.716
End-systolic length of the right atrium (mm)		71.2±7.3	74.5±10.9	0.625
End-diastolic length of the right ventricle (mm)		39.9±4.8	41.7±5.1	0.886
Pulmonary hypertension (mmHg)		66.5±9.6	69.7±11.8	0.686
Ejection fraction (%)		51.2±6.7	49.5±7.7	0.837
Ejection fraction <35%		0	0	
Atrial fibrillation		12	14	0.787
Left atrial thrombosis		2	4	0.556
Hypertension		7	10	0.731
Diabetes mellitus		5	8	0.607
Hyperlipidemia		7	8	0.832
Aortic disease		0	0	
Liver insufficiency		0	0	
Renal insufficiency		0	0	
Pulmonary infection		4	7	0.535
New cerebral infarction		0	0	
New cerebral hemorrhage		0	0	

BMI=body mass index; NYHA=New York Heart Association

The inclusion criteria were: patients presenting with isolated mitral valve lesions and/or tricuspid valve lesions were selected for both groups. The exclusion criteria were: 1) patients with concurrent surgical treatment of aortic or coronary artery disease; 2) patients with potential surgical complications, such as obesity, severe chest deformity, and history of right chest surgery; 3) patients with femoral arteriovenous malformation; 4) patients with severe valvular heart disease whose cardiac structure form had seriously changed with left ventricular end-diastolic diameter >70 mm or ejection fraction (EF) <35%; 5) patients with a huge left or right atrium, which needed left or right atrial reduction in the same period; 6) patients in poor general condition, accompanied by multiple organ failure; 7) patients with New York Heart Association (NYHA) class grade IV heart failure, with no improvement after treatment; and 8) patients who underwent mitral repair.

The left ventricular EF of all patients was measured by M-mode echocardiography. Left and right ventricular diameter were measured from the anteroposterior diameter of the heart chambers by the level of the left ventricular long axis. The end-diastolic lengths of the right ventricle and the left ventricle and the end-systolic lengths of the right atrium and the left atrium were also measured by the apical four-chamber view. We used the pressure half-time method to estimate the mitral valve area, and the proximal isovelocity surface area method to estimate valve regurgitation. All TTE were performed by two independent sonographers with 20 years of experience. There were no significant differences in inter- and intraobserver variability.

This study was approved by the ethics committee of Fujian Medical University, China, and adhered to the Declaration of Helsinki. Additionally, written informed consent was acquired from the patients or the patient's relatives.

### Minimally Invasive Video-assisted Mitral Valve Replacement with a Small Incision (4-6 cm) in the Right Chest

All patients in this group underwent intravenous and inhalation anesthesia with single-lumen endotracheal intubation. The patient was placed in the supine position and tilted (30º) to the left with a slight elevation of the right side using a pad. A 3-cm longitudinal incision was made in the right groin area to expose the femoral artery and vein, on which a purse-string suture was placed individually (incision 1). A 4-6-cm curved incision was made along with the lower margin of the right breast to gain access to the thoracic cavity via the fourth intercostal space (incision 2). A rib spreader was used to expand the intercostal space. A 2-cm right axillary incision was made to gain access to the thoracic cavity via the second intercostal space (incision 3). This incision was used to place the superior vena cava cannula, superior vena cava occlusion tape, and ascending aorta cross-clamp. A 2-cm incision was made at the right mid axillary line in the fourth or fifth intercostal space to gain access to the thoracic cavity and was used to place the thoracoscope and left ventricular vent tube (incision 4). The pericardium was incised in the site 1 cm anterior to the phrenic nerve, and the aortic root was exposed. The pericardium was discontinued with 5-7 stitches. The traction lines above the midpoint of the pericardium in front of the phrenic nerve were drawn from incision 3, and the other traction lines were drawn from incision 4 to lift the pericardium. Purse-string sutures were placed in the root of the superior vena cava and aorta. Systemic heparinization was achieved, and the femoral artery and vein were cannulated with a femoral artery cannula (#18-20) and a femoral venous cannula (#24-26) (from Medtronic), respectively. After initiation of the bypass, a domestic right-angle superior vena cava cannula (#26-28) was inserted into the root of the superior vena cava. Following the full bypass, the superior and inferior vena cava were clamped, and occlusion tape was introduced out through incision 2. The aortic root was sutured with a double needle with gasket loading out through the incision, and the cardioplegia cannula was inserted. When the body temperature dropped to 32ºC, the ascending aorta was clamped. Antegrade cold blood cardioplegic solutions were administered. The left ventricle vent was started, the right atrium was opened, and the atrial septum was incised. Three sutures were used to hang the anterior incisional edge of the atrial septum and were sutured to the site at the para-sternum. One suture was used to hang the posterior incisional edge of the atrial septum and was sutured to the pericardium. It was explored whether a thrombus was in the left atrial appendage, and if so, the patient was surgically treated for left atrial thrombus, and the left atrial appendage was closed. The mitral valve was then visualized. The lesioned valve was excised, and valve replacement was performed via an intermittent suture method (11 St. Jude Mechanical Valves of 25~29#, six GK Mechanical Valves of 25~29#, and three Edward Biological Valves of 27#). GK Mechanical Valves were fabricated by Beijing Sida Medical Equipment Co. Ltd. and implanted in the supra annular position. Continuous sutures were used to close the atrial septal incision and the right atrial incision. After rewarming, the clamps of the ascending aorta and the superior and inferior vena cava were removed. The patient was eventually weaned off the CPB. A chest tube was placed via incision 4 for closed-chest drainage^[8,20]^.

### Conventional Mitral Valve Replacement

All patients in this group underwent intravenous and inhalation anesthesia with single-lumen endotracheal intubation. A 20-cm midline incision of the sternum was used for cannulation of the aorta and the superior and inferior venae cava for establishment of CPB. During surgery, the mitral valve was exposed via the right atrium-septum approach. It was explored whether there was a thrombus in the left atrial appendage, and if so, the patient was surgically treated for left atrial thrombus, and the left atrial appendage closed. The lesioned mitral valve was removed, and an artificial mitral valve was placed with intermittent sutures (14 St. Jude Mechanical Valves of 25~29#, eight GK Mechanical Valves of 25~29#, and three Edwards Biological Valves of 25~27#). Pericardial and mediastinal drainage tubes were placed and allowed to drain through the lower site of the incision.

In these two groups, patients who had combined tricuspid regurgitation and left atrial thrombosis were treated with tricuspid valve repair and left atrial thrombectomy during the same period. If left atrial thrombosis or/and atrial fibrillation occurred, the left atrial appendage needed to be closed. All patients with moderate or severe tricuspid valve regurgitation were treated with Devega angioplasty. All the procedures were performed by the same team of surgeons.

### Statistical Analysis

The Statistical Package for the Social Sciences (SPSS) software, version 19.0, was used for statistical analysis. Quantitative data with a normal distribution are expressed as the mean ± standard deviation (x±s). The independent samples t-test was used to compare between-group differences. Qualitative data were compared using the χ^2^ test. A P<0.05 was considered significantly different.

## RESULTS

Table 1 shows that the preoperative data of both groups, including age, sex, quality index, cardiac function, type and degree of valvular lesions, transthoracic echocardiographic results, and complications, had no significant differences. The two groups were comparable.

The operation was successfully completed in both groups. There was no significant difference in the operating time, aortic occlusion clamping time, or CPB time between the two groups. The length of incision in group A was obviously shorter than in group B, and the difference was significant (Table 2). Group A had shorter postoperative analgesia time, shorter postoperative hospital length of stay, lower drainage volume, and lower blood transfusion volume than group B, and these differences were significant. There were no significant differences in postoperative mechanical ventilation time, postoperative intensive care unit time, and hospital costs (Table 2).

**Table 2:** Comparison of patients' clinical data between the two groups.

Item	Thoracoscopy-assisted group	Conventional group	*P*-value
Operative time (min)	148.2±30.9	151.5±24.4	0.768
Aortic occlusion clamping time (min)	50.1±12.5	43.6±9.7	0.815
Cardiopulmonary bypass time (min)	73.2±13.8	68.7±14.9	0.713
Incision's length (cm)	4.8±1.0	19.6±1.4	0.001
Postoperative mechanical ventilation time (h)	21.4±6.1	23.1±7.8	0.677
Postoperative intensive care unit time (days)	2.2±1.4	2.7±1.7	0.933
Drainage (ml)	210.3±60.8	510.8±71.3	0.011
Blood transfusion volume (ml)	382.2±56.4	718.5±117.8	0.009
Postoperative analgesia time (h)	22.1±5.6	48.5±6.4	0.023
Postoperative hospital length of stay (days)	10.5±2.9	14.2±2.1	0.047
Hospital costs (10000RMB*)	9.1±1.1	8.7±1.7	0.958

* Costs in renminbi (the Chinese currency)

There were no complications, such as heart failure, malignant arrhythmia, cerebral infarction, intracerebral hemorrhage, delayed awakening, perivalvular leakage, blockage, or secondary valve replacement. The incidence rate of liver insufficiency, acute renal insufficiency, pulmonary insufficiency, and gastrointestinal bleeding was not significantly different between the two groups. There were significantly more patients with poor wound healing and pulmonary infection in group B than in group A. Fewer patients underwent re-operation for bleeding in group A than in group B, and the number of patients with pneumothorax and subcutaneous emphysema in group A was greater than in group B (Table 3).

**Table 3:** Comparison of postoperative complications between the two groups.

Item	Thoracoscopy-assisted group	Conventional group	*P*-value
Death	0	0	
Cerebral infarction	0	0	
Cerebral hemorrhage	0	0	
Recovery delay	0	0	
Heart failure	0	0	
Malignant arrhythmia	0	0	
Liver insufficiency	1	1	
Acute renal insufficiency	2	3	0.633
Pulmonary insufficiency	1	1	
Gastrointestinal bleeding	1	1	
Pulmonary infection	4	13	0.028
Reoperation for bleeding	0	3	
Poor wound healing	1	7	0.045
Pneumothorax	4	0	0.019
Subcutaneous emphysema	3	0	0.045
Perivalvular leakage	0	0	
Mechanical valve flap	0	0	

The preoperative renal function of both groups was slightly damaged, as the creatinine value in the two groups was higher than the normal limit, and the total glomerular filtration rate was lower than 60 ml/min; there was no significant difference between the two groups. The incidence of postoperative renal insufficiency did not significantly differ between the video-assisted group and the conventional group, which indicated that the video-assisted operation did not increase the risk of renal insufficiency (Tables 3 and 4).

**Table 4:** Comparison of preoperative and postoperative renal function between the two groups.

Item	Thoracoscopy-assisted group	Conventional group	*P*-value
Preoperative creatinine (umol/L)	98.6±12.3	105.7±15.4	0.675
Preoperative kidney GFR (ml/min)	54.4±6.3	51.7±7.6	0.802
Creatinine in the 1^st^ postoperative day (umol/L)	221.4±21.5	239.6±32.1	0.823
Creatinine in the 3^rd^ postoperative day (umol/L)	146.8±31.4	151.5±44.9	0.875
Creatinine in the 7^th^ postoperative day (umol/L)	103.4±12.7	108.9±15.1	0.916
Creatinine in the 10^th^ postoperative day (umol/L)	99.7±14.2	100.5±13.4	0.936

GFR=glomerular filtration rate

The median duration of follow-up was 32 months (range: 18-60 months). No patient was lost to follow-up. There were no prosthetic valve-related complications or sudden death during the follow-up period. In addition, there were no significant differences between the two groups in left ventricular end-diastolic diameter, left atrial diameter, right atrial diameter, right ventricular end-diastolic diameter, EF, pulmonary arterial pressure, maximum mitral transvalvular pressure, or heart function after the operation, which illustrated that there was no significant difference in efficacy between the two groups (Table 5).

**Table 5:** One-year follow-up echocardiographic data comparison between two group of patients.

Item	Thoracoscopy-assisted group	Conventional group	*P*-value
End-systolic length of the left atrium (mm)	45.6±5.4	47.4±6.5	0.877
End-diastolic length of the left ventricle (mm)	53.4±4.3	54.2±5.5	0.923
End-systolic length of the right atrium (mm)	41.1±5.2	45.6±6.7	0.881
End-diastolic length of the right ventricle (mm)	34.4±4.1	36.9±5.1	0.867
Ejection fraction (%)	58.8±7.3	55.4±8.4	0.812
Pulmonary hypertension (mmHg)	30.2±9.9	27.5±11.2	0.654
Max MV PG (mmHg)	10.3±1.8	11.5±2.3	0.956
Mechanical valve flap	0	0	
Perivalvular leakage	0	0	

MV=mitral valve; PG=peak gradient

## DISCUSSION

The World Health Organization defines older persons as over 65 years. Because elderly have their own characteristics, CPB and cardiac arrest are more likely to cause more severe injury^[21]^. Anderson AJ retrospectively analyzed the clinical data of 265 patients over 70 years old who were undergoing CPB. The results showed that the longer the CPB time, the higher the perioperative mortality. Compared with patients with a CPB time shorter than 75 minutes, the perioperative risk of death for patients with a CPB time longer than 75 minutes increased by 3.2 times^[3]^. Therefore, shortening the cardiac arrest and CPB times should be prioritized in mitral valve replacement operations of elderly patients, and myocardial protection should be simultaneously strengthened.

Video-assisted mitral valve replacement via a small incision in the right chest in this study was performed under the direct view of the small incision and was assisted by thoracoscopy with a 4-6-cm incision. The operation can be performed by conventional surgical instruments, and it does not require long thoracoscopic instruments, which greatly decreases the difficulty of the operation and shortens the operative time. In this study, the duration of aortic occlusion and CPB in the video-assisted group did not significantly differ from that in the conventional group. This outcome indicates that the video-assisted operation does not increase the injury due to cardiac arrest and CPB. Video-assisted mitral valve replacement via a small incision in the right chest has advantages of both a small incision and small wound, which can shorten the time of hemostasis, closure of the chest, and suturing of the incision^[22]^.

The CPB pipeline and minimally invasive incision will directly affect exposure of the surgical visual field and operation space, increasing the difficulty and risk of the operation. Our hospital has improved and optimized the CPB method, and we have adopted femoral artery and femoral vena cava cannula and right-angle superior vena cava cannula (with ascending aortic clamping forceps drawn from a small hole, incision 3). Compared with the CPB method of total thoracoscopic surgery (jugular vena cava cannula), this CPB method does not affect the surgical visual field. It can also ensure drainage and provide a stable state of CPB without the use of a negative pressure drainage device to assist circulation, which can reduce damage to blood cells. The key to the mitral valve replacement operation is how to expose the mitral valve in the endoscopic operation. The anterior edge of the atrial septal incision was lifted by three traction wires in perforation of the sternum, and the posterior edge of the atrial septal incision was lifted by one traction wire with the pericardium; then, the mitral valve was clearly exposed. In the video view, this simple modified method can expose the mitral valve and its surrounding structures more clearly than the conventional operation.

Patients aged over 65 years who received excessive amounts of infused blood products are prone to complications, such as perfusion lung, pulmonary edema, etc^[23]^. The coagulation and tissue mechanisms are significantly more impaired in elderly patients than in younger patients. Additionally, because of osteoporosis, the elderly are prone to greater blood leakage from the sternum, the posterior sternal wound, and the area of steel wire behind the sternum than younger patients. The video-assisted operation does not require to split the sternum; thus, bleeding of the surgical wound is markedly reduced, bleeding is easier to stop, and the need for blood products decreases accordingly.

The postoperative pulmonary insufficiency is the most important complication and independent influencing factor for death in heart valve replacement in elderly patients^[24,25]^. The video-assisted operation confers advantages of a small incision, no need to split the sternum, and good thoracic stability. In addition, these patients are able to cough better, expectorate, and get out of bed earlier, with less pain. The incidence of pulmonary infection is obviously reduced, which helps to speed up postoperative physical recovery and shorten the hospital length of stay. With a longer incision and the use of wire and bone wax, the risk of foreign body rejection is higher in the conventional operation. The subcutaneous tissue in the middle of the sternum is minimal; thus, the risks of bad wound healing and fat liquefaction are increased. During the operation in the video-assisted group, the right lung should be squeezed, which may injure the lung tissue, especially in the process of thoracotomy and perforation. The suture needle may also injure the right lung when the rib is closed and sutured. Pneumothorax and subcutaneous emphysema may also occur if the tube hole is not closed properly while pulling out the drainage tube.

Renal insufficiency is a common and severe postoperative complication in mitral valve lesion elderly patients and is an independent predictor of early death after cardiac valve surgery in the elderly^[24,25]^. Before the operation, we actively treated patients to improve cardiac function and avoided renal-damaging drugs to protect their renal function and improve it. For patients with preoperative renal insufficiency, we monitored the changes in creatinine and urine more frequently. If creatinine continues to increase without urine, hemodialysis should be performed as soon as possible.

The echocardiographic data after one-year follow-up showed that there were no significant differences in the recovery of cardiac function between the two groups, but compared with before the operation, cardiac structure and cardiac function were improved. This outcome indicated that both operation methods had good curative effects at the early follow-up, and these curative effects were similar. Thus, the choice of operation method does not affect the early clinical effect.

There are several limitations to this study. This was a single-center retrospective study with a small sample size and a selective bias in cases. Although the small sample size had some effect on statistical power, our results supported our conclusions to some extent. We hope to perform a prospective, multicenter, randomized controlled trial in the future. Additionally, the follow-up time of this study was short, and a longer follow-up needs to be completed.

## CONCLUSION

Applications of minimally invasive video-assisted mitral valve replacement with a small incision in the right chest in patients aged over 65 years had the same clinical efficacy and safety as the conventional method. With advantages of a small incision, less bleeding, less pain, and faster recovery, minimally invasive video-assisted mitral valve replacement is a good surgical method for patients aged over 65 years.

**Table:** 

Author's roles & responsibilities	
QC	Substantial contributions to the conception or design of the work; drafting the work or revising it critically for important intellectual content; final approval of the version to be published
LLY	Final approval of the version to be published
QLZ	Final approval of the version to be published
HC	Final approval of the version to be published
LWC	Final approval of the version to be published
ZYH	Substantial contributions to the conception or design of the work; drafting the work or revising it critically for important intellectual content; final approval of the version to be published

## References

[1] Barnett SD, Halpin LS, Speir AM, Albus RA, Akl BF, Massimiano PS (2003). Postoperative complications among octogenarians after
cardiovascular surgery. Ann Thorac Surg.

[2] Zhang Baoren, Xu Zhiyun, Zou Liangjian, Mei Ju, Hao Jiahua, Xing Jianzhou (2004). Analysis of 265 cases of mitral valve replacement in elderly
patients. Chin J Clin Thorac Cardiovasc Surg.

[3] Anderson AJ, Barros Neto FX, Costa Mde A, Dantas LD, Hueb AC, Prata MF (2011). Predictors of mortality in patients over 70 years-old undergoing
CABG or valve surgery with cardiopulmonary bypass. Rev Bras Cir Cardiovasc.

[4] Davis 3rd WJ, Vaynblat M, Chiavarelli M, Shah P, Fazylov R, Zisbrod Z (2004). Open heart surgery in patients 85 years and older. J Card Surg.

[5] Nair CK, Biddle WP, Kaneshige A, Cook C, Ryschon K, Sketch Sr MH (1992). Ten-year experience with mitral valve replacement in the
elderly. Am Heart J.

[6] Bastidas JG, Razzouk AJ, Hasaniya NW, Bailey LL (2013). Left thoracotomy: an ideal approach for mitral valve replacement
in patient with severe chest wall deformity. Ann Thorac Surg.

[7] Saito S, Tsukui H, Iwasa S, Umehara N, Tomioka H, Aomi S (2016). Bileaflet mechanical valve replacement: an assessment of outcomes
with 30 years of follow-up. Interact Cardiovasc Thorac Surg.

[8] Cao H, Chen Q, Li QZ, Chen LW, Zhang GC, Chen DZ (2014). A clinical study of thoracoscopy-assisted mitral valve
replacement concomitant with tricuspid valvuloplasty, with domestically
manufactured pipeline products for cardiopulmonary bypass. J Cardiothorac Surg.

[9] Cohn LH, Adams DH, Couper GS, Bichell DP, Rosborough DM, Sears SP (1997). Minimally invasive cardiac valve surgery improves patient
satisfaction while reducing costs of cardiac valve replacement and
repair. Ann Surg.

[10] Cosgrove 3rd DM, Sabik JF, Navia JL (1998). Minimally invasive valve operations. Ann Thorac Surg.

[11] Gammie JS, Zhao Y, Peterson ED, O’Brien SM, Rankin JS, Griffith BP (2010). Less-invasive mitral valve operations: trends and outcomes from
the society of thoracic surgeons adult cardiac surgery
database. Ann Thorac Surg.

[12] Ito T, Maekawa A, Hoshino S, Hayashi Y, Sawaki S, Yanagisawa J (2017). Three-port (one incision plus two-port) endoscopic mitral valve
surgery without robotic assistance. Eur J Cardiothorac Surg.

[13] Schmitto JD, Mokashi SA, Cohn LH (2011). Past, present, and future of minimally invasive mitral valve
surgery. J Heart Valve Dis.

[14] Zhao G, Gao J, Liu Y, Gu S, Guo Y, Xie B (2017). Two-incision totally thoracoscopic approach for mitral valve
replacement. Int Heart J.

[15] Qiao Y, An G, Chen G, Zheng S, Ni L, Wang W (2014). Minimally invasive video-assisted double-valve replacement
through right anterolateral Minithoracotomy. Heart Lung Circ.

[16] Zhai J, Wei L, Huang B, Wang C, Zhang H, Yin K (2017). Minimally invasive mitral valve replacement is a safe and
effective surgery for patients with rheumatic valve disease: a retrospective
study. Medicine (Baltimore).

[17] Cao C, Gupta S, Chandrakumar D, Nienaber TA, Indraratna P, Ang SC (2013). A meta-analysis of minimally invasive versus conventional mitral
valve repair for patients with degenerative mitral disease. Ann Cardiothorac Surg.

[18] Mishra Y, Sharma M, Bapna R, Malhotra R, Mehta Y, Sharma KK (2002). Minimally invasive mitral valve surgery. Indian Heart J.

[19] Suri RM, Schaff HV, Meyer SR, Hargrove 3rd WC (2009). Thoracoscopic versus open mitral valve repair: a propensity score
analysis of early outcomes. Ann Thorac Surg.

[20] Zhang QL, Chen Q, Lin ZQ, Yu LL, Lin ZW, Cao H (2018). Thoracoscope-assisted mitral valve replacement with a small
Incision in the right chest: a chinese single cardiac center
experience. Med Sci Monit.

[21] Stallwood MI, Grayson AD, Mills K, Scawn ND (2004). Acute renal failure in coronary artery bypass surgery:
independent effect of cardiopulmonary bypass. Ann Thorac Surg.

[22] Schroeyers P, Wellens F, De Geest R, Degrieck I, Van Praet F, Vermeulen Y (2001). Minimally invasive video-assisted mitral valve repair: short and
mid-term results. J Heart Valve Dis.

[23] Koch CG, Li L, Duncan AI, Mihaljevic T, Cosgrove DM, Loop FD (2006). Morbidity and mortality risk associated with red blood cell and
blood-component transfusion in isolated coronary artery bypass
grafting. Crit Care Med..

[24] McLean RC, Briggs AH, Slack R (2011). Perioperative and long-term outcomes following aortic valve
replacement: a population cohort study of 4124 consecutive
patients. Eur J Cardiothorac Surg.

[25] Collart F, Feier H, Kerbaul F, Mouly-Bandini A, Riberi A, Mesana TG (2005). Valvular surgery in octogenarians; operative risks factors
evaluation of Euroscore and long term results. Eur J Cardiothorac Surg.

